# A Monitoring Device and Grade Prediction System for Grain Mildew

**DOI:** 10.3390/s24206556

**Published:** 2024-10-11

**Authors:** Lei Xu, Yane Li, Xiang Weng, Jiankai Shi, Hailin Feng, Xingquan Liu, Guoxin Zhou

**Affiliations:** 1 College of Mathematics and Computer Science, Zhejiang A&F University, Hangzhou 311300, China; xulei@stu.zafu.edu.cn (L.X.); yaneli@zafu.edu.cn (Y.L.); woodweng@zafu.edu.cn (X.W.); 2 Key Laboratory of Forestry Intelligent Monitoring and Information Technology of Zhejiang Province, Hangzhou 311300, China; 3 China Key Laboratory of State Forestry and Grassland Administration on Forestry Sensing Technology and Intelligent Equipment, Hangzhou 311300, China; 4 School of Computer Science and Technology, Hangzhou Dianzi University, Hangzhou 310018, China; 5 College of Food and Health, Zhejiang A&F University, Hangzhou 311300, China; liuxq@zafu.edu.cn; 6 National Grain Industry (High-Quality Rice Storage in Temperate and Humid Region) Technology Innovation Center, Zhejiang A&F University, Hangzhou 311300, China; 7 College of Modern Agriculture, Zhejiang A&F University, Hangzhou 311300, China; gxzhou211@foxmail.com

**Keywords:** mildew, rice, monitoring device, CNN–LSTM–Attention, time series prediction

## Abstract

Mildew infestation is a significant cause of loss during grain storage. The growth and metabolism of mildew leads to changes in gas composition and temperature within granaries. Recent advances in sensor technology and machine learning enable the prediction of grain mildew during storage. Current research primarily focuses on predicting mildew occurrence or grading using simple machine learning methods, without in-depth exploration of the time series characteristics of mildew process data. A monitoring device was designed and developed to capture high-quality microenvironment parameters and image data during a simulated mildew process experiment. Using the “Yongyou 15” rice varieties from Zhejiang Province, five simulation experiments were conducted under varying temperature and humidity conditions between January and May 2023. Mildew grades were defined through manual analysis to construct a multimodal dataset for the rice mildew process. This study proposes a combined model (CNN–LSTM–A) that integrates convolutional neural networks (CNN), long short-term memory (LSTM) networks, and attention mechanisms to predict the mildew grade of stored rice. The proposed model was compared with LSTM, CNN–LSTM, and LSTM–Attention models. The results indicate that the proposed model outperforms the others, achieving a prediction accuracy of 98%. The model demonstrates superior accuracy and more stable performance. The generalization performance of the prediction model was evaluated using four experimental datasets with varying storage temperature and humidity conditions. The results show that the model achieves optimal prediction stability when the training set contains similar storage temperatures, with prediction accuracy exceeding 99.8%. This indicates that the model can effectively predict the mildew grades in rice under varying environmental conditions, demonstrating significant potential for grain mildew prediction and early warning systems.

## 1. Introduction

The introduction of modern technologies to grain production has resulted in higher quality and yield. However, the development of static grain storage technologies has lagged, failing to meet the post-harvest storage needs of grains [1]. Mildew is one of the main causes of grain loss during storage, affecting not only the quality of the grain but also posing significant food safety risks due to the toxins produced by molds [2]. Grain storage managers can quickly and easily detect mildew using hyperspectral imaging [3] or an electronic nose [4], but only after mildew and grain loss has occurred. Therefore, the early prediction and grade recognition of mildew are quite necessary for the prevention and control of mildew and the safe storage of rice. Currently, experts and scholars at home and abroad are conducting extensive research in the field of grain mildew prediction using IoT technology, sensor technology, and machine learning methods.

By detecting the volatile organic compound (VOC) gases produced during the mildew formation process, such as CO_2_ produced by respiration, hydroxyl groups, aldehyde groups, and sulfides produced by grain metabolism and microbial activity, non-destructive monitoring of grain mildew can be achieved [4,5]. Temperature and humidity are the main factors influencing VOC production, and the metabolism of microorganisms such as molds is significantly correlated with the release rate of CO_2_. Ramacandran [6] reviewed the role of CO_2_ in the grain storage environment and the potential for early mildew detection using CO_2_ sensing and monitoring technology, analyzing the production and movement processes of CO_2_ in grain silos, and providing guidance for ventilation strategies. In addition to humidity, temperature, and CO_2_ content in the grain silo environment, the internal temperature and moisture content of the grain are also key indicators for monitoring grain condition. Real-time monitoring with sensors can detect grain anomalies at the early stages of mildew formation, allowing timely measures to be taken, and effectively reducing mildew risk and economic losses [7,8,9]. However, simply identifying whether mildew has occurred is insufficient to guide precise prevention and control measures in grain silos. Furthermore, using uniform treatment for different degrees of mildew can lead to resource wastage and increased storage costs. Therefore, scholars have begun to explore the graded prediction of grain mildew, but most existing studies use simple machine-learning algorithms for mildew grading. Wang et al. [7] used colorimetric sensor technology and applied various linear (KNN, LDA) and nonlinear (ELM, SVM) chemometric methods for high-precision qualitative identification of wheat mildew grades but only used the number of days to indicate the degree of mildew without providing a classification of mildew grades. Deng et al. [10] researched mildew prediction technology based on BP neural networks but their modeling required a large training sample set and faced low accuracy with small samples. Yuan et al. [11] used a support vector machine (SVM) algorithm to establish a prediction classification model for rice and wheat mildew to determine whether mildew would occur under given moisture, temperature, and storage time conditions, achieving an average prediction classification accuracy of 90%. Later, Yuan et al. [12] improved and established a new model (FOA-SVM) based on the fruit fly optimization algorithm, which can automatically search for parameters and adjust its parameters automatically when the dataset changes to find the best results, demonstrating strong self-adjustment capabilities. The accuracy of predicting stored grain mildew grades using the FOA-SVM algorithm is 85.40%. Yang et al. [13] combined hyperspectral imaging with the deep stacked sparse autoencoder (SSAE) algorithm to identify mildew grades in corn kernels, initially introducing deep learning algorithms into mildew identification research and achieving a recognition accuracy of 96% in the test set. However, current research only focuses on the grain mildew state at a single point in time [11,12], neglecting the temporal variation characteristics of the mildew process, and the accuracy, stability, and reliability of the models need to be improved.

Time series prediction algorithms have significant advantages in exploring future trends in data. Compared to traditional machine learning algorithms, deep learning-based time series prediction algorithms offer stronger generalization capabilities and higher prediction accuracy [14,15,16]. They have been widely used in fields such as finance [17], meteorology [18], transportation [19], forestry [20] and healthcare [21]. In the agricultural domain, researchers have also started to introduce time series prediction algorithms to address problems in agricultural production. Lu et al. [22] developed an Informer-based model for predicting heavy metal (HM) safety risks in rice, focusing on supervision and early warning in areas contaminated by heavy metals (HMs). The proposed method achieved prediction accuracies of 99.17%, 91.77%, and 91.33% for low, medium, and high-risk levels, respectively. Xu et al. [23] developed a deep learning model based on RGB and hyperspectral images for the rapid classification of tea sooty mildew. The CARS–LSTM model showed the best overall performance in grading the severity of tea sooty mildew, with an accuracy of 95%. Chen et al. [24] used long short-term memory (LSTM) and recurrent neural networks (RNN) to predict crop diseases and pests, demonstrating good performance in predicting pest occurrences in cotton fields, with an area under the curve (AUC) of 0.95. Akkem et al. [25] reviewed research on time series analysis algorithms in agriculture, especially in crop yield prediction. These studies demonstrate the significant potential of time series prediction algorithms in agriculture. However, there has been little research on predicting grain mildew using time series prediction algorithms. Grain mildew, as a typical temporal variation process, has data with significant temporal characteristics. Using deep learning time series prediction algorithms to fully explore and utilize the temporal features of mildew data can help achieve more accurate and reliable grain mildew predictions.

Therefore, this study aims to explore the relationships between grain condition parameters and the evolution of mildew conditions using deep learning time series prediction algorithms to predict mildew severity levels. The core issue is how to simulate the grain mildew process under different storage conditions, collect corresponding data, define mildew severity levels, and improve the accuracy of mildew severity level predictions. To date, no one has conducted research in this application area. In this study, taking rice as the research object, a software and hardware system for multi-dimensional data collection and storage of grain conditions was first designed and developed. This system can continuously monitor environmental temperature and humidity, oxygen concentration, carbon dioxide concentration, and internal temperature and moisture of rice during the mildew process. Simultaneously, it collects images of the rice mildew process and defines mildew severity levels through manual analysis, ultimately constructing a multimodal dataset of the rice mildew process. Then, the CNN–LSTM–A improved algorithm was used to extract temporal features from the mildew process data and establish a rice mildew severity level prediction model.

The contributions of this work mainly include the following three points: Firstly, this study innovatively combines rice mildew process images with manual sensory analysis to define mildew severity levels, designs and conducts mildew process simulation experiments and constructs a multimodal dataset of the rice mildew process. Secondly, this study is the first to propose a CNN–LSTM–A improved algorithm-based grain mildew severity level prediction model. This model effectively addresses the problem of predicting rice mildew severity levels under different storage conditions, thus aiding in the management of rice storage to reduce mildew occurrence. Lastly, the improved model proposed in this study achieved prediction accuracies of up to 99.84% and recall rates of up to 99.83% on different datasets. Additionally, the performances of the LSTM, LSTM–A, CNN–LSTM, and CNN–LSTM–A models were compared. The experimental results indicate that the proposed model has high prediction accuracy and good generalization performance.

**Figure 1:**
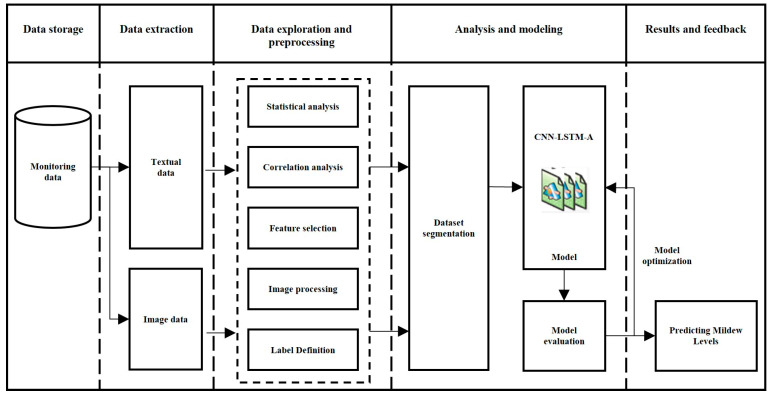
Flowchart of rice mildew grade prediction.

By using this rice mildew prediction method to predict the probability of mildew in grain warehouses, managers can pay more attention to grain warehouses when the probability of mildew is high. This provides a new solution for precise mildew prevention and control in grain warehouses. The process of rice mildew severity level prediction in this study is shown in Figure 1.

## 2. Materials and Methods

### 2.1. Experimental Setup

**Figure 2:**
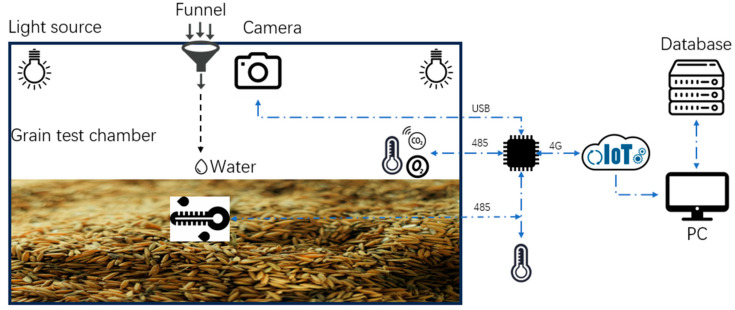
System block diagram of grain mildew process monitoring device.

This experiment simulates the mildew growth process during rice storage, using the “Yongyou 15” rice variety from Zhejiang Province, China. The 2.5 kg rice samples were placed in an experimental box of 60 cm × 42 cm × 33 cm for simulated storage, and a grain mildew growth monitoring device was used to obtain microenvironment parameters and mildew images at various stages of the rice mildew growth process. The system block diagram of the device is shown in Figure 2.

The grain mildew growth monitoring device consists mainly of an environmental parameter collection unit, an image acquisition unit, a data processing device, and a computer. The environmental parameter collection unit includes temperature and humidity sensors, grain temperature and moisture sensors, carbon dioxide sensors, and oxygen sensors. The rice images collected by the image acquisition unit are 1280 × 720 pixels. The specific parameters of each sensor are shown in Table 1.

**Figure 3:**
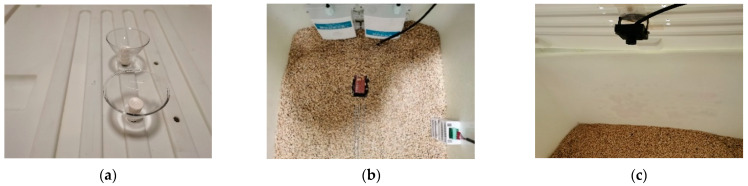
Schematic diagram of data acquisition in the experimental chamber: (**a**) funnel opening for water filling, (**b**) microenvironment sensor deployment in the experimental box, (**c**) top camera.

To accelerate the mildew growth of the rice, the experiment simulates the mildew growth process after the rice becomes damp during storage by using a spray method to moisten a part of the rice. Through the funnel on the cover of the experimental box, 5 mL of deionized water is applied to the rice every 12 h, and environmental parameters inside and outside the experimental box are collected at 1 min intervals, while mildew image data are collected at 10 min intervals. Environmental parameters inside the experimental box, such as oxygen, carbon dioxide, temperature, and humidity, are collected by sensors placed inside the box. Rice temperature and moisture are collected by placing sensors 3–5 cm inside the rice, and mildew images are taken from about 30 to 35 cm above the rice surface. External sensors are used to collect outside temperature and humidity. The installation positions of the data collection devices inside and outside the experimental box are shown in Figure 3.

**Figure 4:**
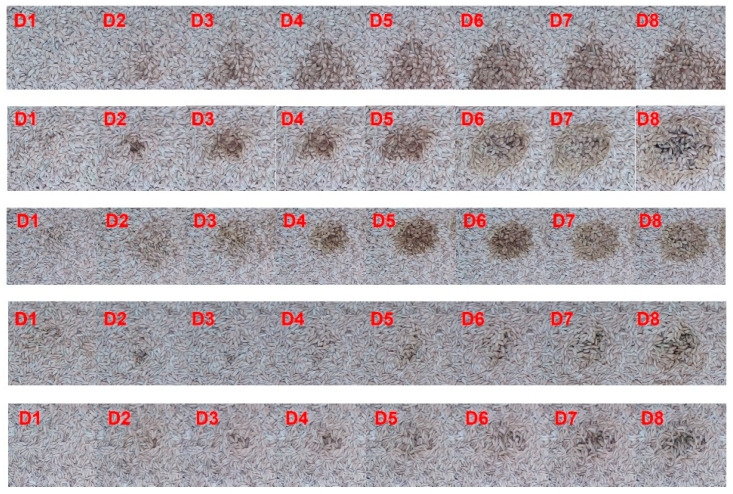
Eight consecutive days image dataset of rice mildew process. Dn denotes the nth day.

In five simulation experiments, images capturing the continuous changes in mildew growth were taken throughout the entire mildew growth process over 8 days. The details are shown in Figure 4.

### 2.2. Data Collection

The rice variety “Yongyou 15” series in Zhejiang Province, China, was used to carry out five simulation experiments in different months. The experiments were carried out successively from January to May 2023, and the difference between different experiments mainly lies in the ambient temperature and humidity. The collection interval of each experiment was 1 min, so a total of 11,492 datasets were formed in each experiment. There were 57,460 datasets in 5 experiments (Table 2). Each dataset contains defining labels for collection time, external temperature and humidity, internal temperature and humidity, grain moisture, grain temperature, internal carbon dioxide content, internal oxygen content, and mildew grade.

**Figure 5:**
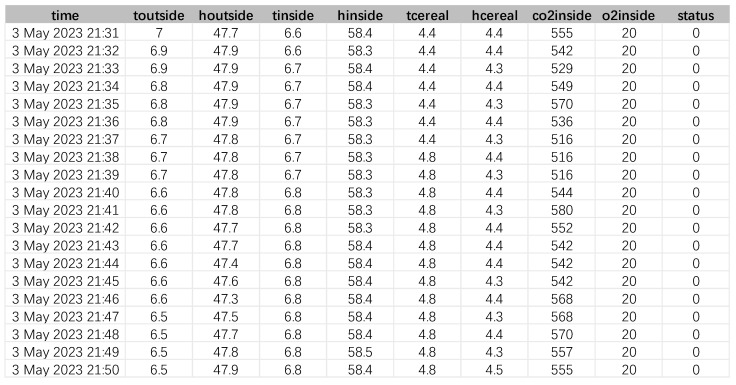
Experimental process dataset.

The dataset contains defining labels for collection time, external temperature and humidity, internal temperature and humidity, grain moisture, grain temperature, internal carbon dioxide content, internal oxygen content, and mildew grade. The experimental process dataset is shown in Figure 5.

### 2.3. Data Preprocessing

First, missing or abnormal samples are eliminated by performing data cleaning. Additionally, due to the different dimensions of different impact factors, the sample data (storage temperature, storage humidity, and other environmental parameters) usually cannot be used directly. The max–min normalization method is employed to standardize the dataset, eliminate the dimensional influence between feature vectors and ensure the running speed of the model.

(1) $$X_{new}=\frac{X-X_{min}}{X_{max}-X_{min}}$$

where X is the original value. $X_{max}$ and $X_{min}$ are the maximum and minimum values of X, respectively. $X_{new}$ indicates the normalized value.

After mapping the data to the [0, 1] space, the dataset of each experiment is divided into the training set and the test set according to the ratio of 8:2. The training set data are used for model training. The test set data are used for model prediction verification.

**Figure 6:**
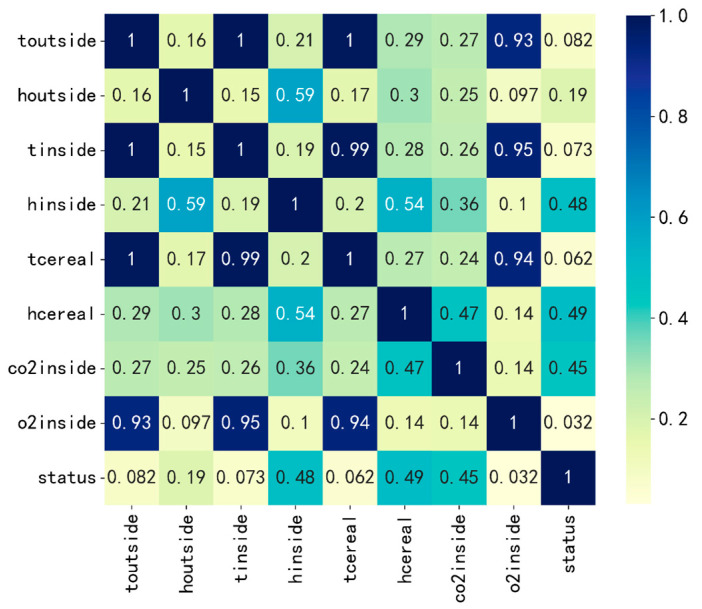
Correlation coefficient between grain parameters and mildew state.

Correlation analysis helps to eliminate data redundancy, improve data quality, and optimize feature sets for modeling. The correlation coefficient between grain condition parameters and mildew state is illustrated in Figure 6, where toutside is the external temperature, houtside is the external humidity, tinside is the internal temperature, hinside is the internal humidity, tcereal is the grain temperature, hcereal is the grain moisture, co2inside is the internal carbon dioxide concentration, o2inside is the internal oxygen concentration, and status indicates mildew grade. According to the correlation coefficient graph, four characteristic variables, namely external humidity, internal humidity, grain moisture, and internal carbon dioxide concentration, were selected as the input of the prediction model. The model output is the prediction result of mildew grade, and the prediction model of grain mildew grade is constructed.

### 2.4. Data Labeling

According to the definition of different stages of rice mildew growth, the mildew condition was analyzed manually using the sensory quality identification method for moldy grain. Previous studies have shown that the dominant fungi in the rice mildew growth process are mainly Aspergillus and Penicillium fungi [5]. Moldy grains are an intuitive manifestation of grain mildew, and the national standard GB 2715-2016 defines them as grains with obvious mildew on the surface, affecting the embryo, endosperm, or cotyledon, rendering them inedible [26]. Sensory detection for grain quality evaluation is fast, simple to operate, and the most widely used method.

Although early or slight mildew growth in rice is not easily detectable, it can be promptly identified and addressed using prior knowledge and computer vision methods based on subtle color changes in the grains [27]. The GB/T 20569-2006 standard for determining the storage quality of rice defines the suitable storage state based on color, categorizing it as normal, basically normal, or yellow, dark gray, brown, or other unacceptable abnormal colors [28]. Referring to LS/T 6132-2018 “Grain and Oil Inspection Storage Fungus Detection Spore Count Method”, the mildew growth stages are classified into safe, critical, and harmful [29]. The fungal types and color states corresponding to each level are shown in Table 3.

**Figure 7:**
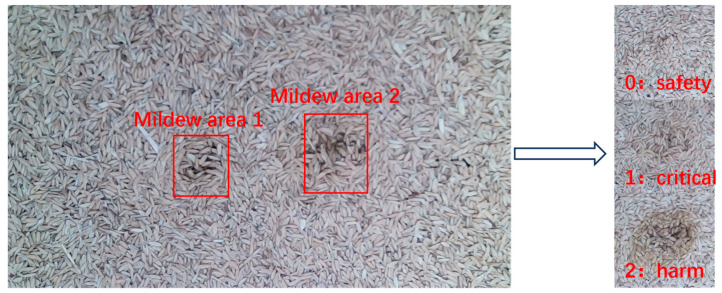
Labeling of rice mildew grade.

Using manual sensory analysis to evaluate and quantify the mildew grades of rice at different stages of mildew growth, the collected rice image data were annotated with mildew grades. In the mildew dataset, the mildew states “safe”, “critical”, and “harmful” are labeled as “0”, “1”, and “2”, respectively. The specific image analysis mildew grade annotation is shown in Figure 7.

### 2.5. Deep Learning Algorithms

#### 2.5.1. Convolutional Neural Networks

Convolutional Neural Networks (CNN) are one of the most important networks in deep learning, designed to extract features from data with a convolutional structure. CNNs have achieved outstanding results in various fields such as computer vision for grading [30], classification [31] and identification [32].

Generally, CNNs consist of an input layer, convolutional layers, pooling layers, fully connected layers, and an output layer. Through the convolutional layers, CNNs can quickly and accurately uncover potential features from the input data. However, the feature maps obtained after convolution contain many features, which can easily lead to overfitting problems [33]. Therefore, to address redundancy, scholars have proposed the pooling operation, also known as down-sampling [34]. Correspondingly, the pooling layer serves to speed up the training of the model and prevent overfitting. By reducing the dimensionality of the extracted features, the amount of training information can be decreased. Adding a pooling layer between two adjacent convolutional layers can improve the training speed of the model.

#### 2.5.2. Long Short-Term Memory

The Long Short-Term Memory (LSTM) model is an extension of the Recurrent Neural Network (RNN) aimed at solving the gradient explosion and vanishing problems of RNNs. LSTM is good at capturing long-term dependencies in time series data and is widely used in various forecasting tasks, such as financial forecasting [35], weather forecasting [36], and sensor data analysis [37].

The LSTM model consists of three gates: the forget gate, the input gate, and the output gate. The forget gate decides whether to retain or delete existing information by passing the current input $x_{t}$ and the previous hidden state $h_{t-1}$ through an activation function (such as sigmoid). The gate output $f_{t}$ is a value between 0 and 1, where 0 indicates the deletion of the learned value and 1 indicates the retention of the value. The output calculation formula is as follows:

(2) $$f_{t}=\sigma (W_{f}\cdot \left[h_{t-1},x_{t}\right]+b_{f})$$

where $b_{f}$ is called a bias value.

The input gate determines that the gate for adding new information to the LSTM memory has two layers. The output value of the input gate is shown in Formulas (3) and (4). The sigmoid layer determines which values need to be updated. The hyperbolic tangential layer generates vectors of new values that are added to memory. Together, these two layers update LSTM memory, forgetting the current value by multiplying the old value and adding the new value.

(3) $$i_{t}=\sigma (W_{i}\cdot \left[h_{t-1},x_{t}\right]+b_{i})$$

(4) $${\tilde{C}}_{t}=tanh(W_{c}\cdot \left[h_{t-1},x_{t}\right]+b_{c})$$

(5) $${\tilde{C}}_{t}=f_{t}*C_{t-1}+i_{t}*{\tilde{C}}_{t}$$

The output gate uses the sigmoid function to determine the part of the LSTM memory that contributes to the output and maps it between −1 and 1 via the nonlinear tanh function.

(6) $$o_{t}=\sigma (W_{o}\cdot \left[h_{t-1},x_{t}\right]+b_{o})$$

(7) $$h_{t}=o_{t}*tanh(C_{t})$$

#### 2.5.3. Attention Mechanism

Adding model parameters can enhance the expressiveness of the model but also lead to information overload. Attention is an important function of human cognition for understanding the complex world, effectively enhancing the efficiency and accuracy of processing perceptual information. The attention mechanism in deep learning learns how to allocate attention weights to the model’s input by automatically modeling and selecting the most relevant information. The weights represent the proportion of corresponding input information, with larger values indicating more useful input information. By computing the attention probability distribution, the model can filter out the most important input information, thereby highlighting these critical pieces to optimize the performance of the deep learning model [38,39].

Niu et al. [40] proposed a standard attention model architecture based on the common core components of most attention models. First, the neural network attention distribution is calculated. Keys (K) are the source data features, and q is the task-related representation vector. Then, the relevance between the query and the keys is computed using a scoring function f, where the energy score e indicates the importance of the query to the key when determining the next output.

(8) e=f(q,K)

Map the energy score e to the attention weight α via the attention allocation function g:

(9) α=g(e)

After the attention weights and values are obtained, the context vector c is computed. The value v is a data feature representation where each element v corresponds to and only corresponds to one K.

(10) $$c=\phi (\left\{\alpha_{i}\right\},\left\{v_{i}\right\})$$

where the function ϕ returns a single vector of the given set of values with its corresponding weight, which is generally achieved by calculating the weighted sum of v.

(11) $$z_{i}=\alpha_{i}v_{i}$$

(12) $$c=\sum_{i=1}^{n}z_{i}$$

where $z_{i}$ is the weighted representation of the element in the value, and n is the dimension of z.

During the training process, the parameters are optimized using a backpropagation algorithm to minimize prediction errors. The optimized attention weight α is automatically adjusted, enabling the model to allocate appropriate attention across different time series, thereby enhancing prediction accuracy.

### 2.6. Structure of CNN–LSTM–A

**Figure 8:**
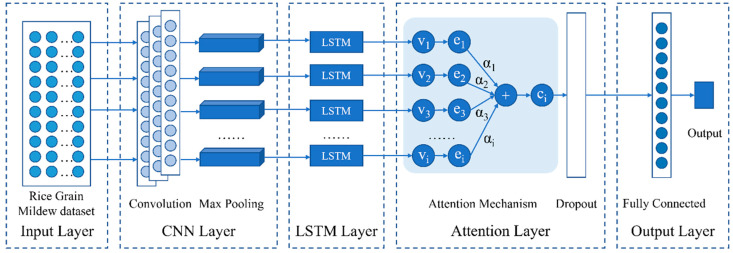
Framework of CNN–LSTM–A prediction algorithm.

This paper proposes a deep learning prediction model based on the combination of LSTM, CNN, and Attention mechanism. The CNN–LSTM–A model consists of five parts: the input layer, the CNN module, the LSTM layer, the attention mechanism layer, and the output layer. This model employs a CNN to extract spatial features from the input data. An LSTM network is used to capture temporal variation patterns. The attention mechanism employs a probability-weighted approach to direct the LSTM network toward the most critical time series for the prediction task, thereby enhancing the model’s accuracy. The structure framework of the CNN–LSTM–A model is shown in Figure 8.

The input layer analyzes the data of the mildew growth process, which is then fed into the CNN layer. By utilizing CNN’s feature extraction capability, deep hidden features can be effectively mined from the raw data, and the filtered new features can be passed as new inputs to the LSTM layer. The LSTM can handle the time series characteristics of the grain condition data, and the LSTM layer can use the hidden state at the last moment of the time series as the output. Combining the respective advantages of CNN and LSTM, attention mechanism further increases the weight ratio of critical information and decreases the weight of non-important information. To reduce the risk of overfitting during training, a Dropout layer is added after the Attention layer, which can randomly mask some neurons during the process, thereby avoiding the model’s dependency on certain features and enhancing the model’s robustness. Using the fully connected layer for weighted summation (for feature synthesis and dimension transformation) enhances prediction accuracy. Finally, the model outputs the predicted mildew grade for the next time point.

### 2.7. Evaluation Metrics

In this study, accuracy (*Acc*), precision (*Pre*), recall (*Rec*), and *F*1 score (*F*1-Score) are used as the performance evaluation metrics of the model. The test set data are utilized to evaluate the performance of the model, which is used to select the optimal model.

Accuracy (*Acc*) refers to the percentage of correctly predicted results out of the total number of samples.

(13) $$Acc=\frac{TP+TN}{TP+TN+FP+FN}\times 100\%$$

The accuracy rate Pre refers to the proportion of positive samples in the predicted positive samples, that is, how many samples in the predicted positive samples are really positive. The accuracy rate is used to reflect the model’s ability to distinguish negative samples. The higher the accuracy rate, the stronger the model’s ability to distinguish negative samples.

(14) $$Pre=\frac{TP}{TP+FP}\times 100\%$$

*TP* (True Positive) indicates an actual positive category and predicts a positive category. *FP* (False Positive) indicates an actual negative category but predicts a positive category. *TN* (True Negative) indicates an actual negative category and predicts a negative category and *FN* (False Negative) indicates an actual positive category but predicts a negative category.

Recall rate Rec refers to the proportion of samples that are predicted to be positive. That is the number of samples predicted to be positive among the really positive samples. The recall rate reflects the model’s ability to distinguish positive samples. The higher the recall rate, the stronger the model’s ability to distinguish positive samples.

(15) $$Rec=\frac{TP}{TP+FN}\times 100\%$$

Pre and *Rec* are contradictory quantities. When *Pre* is high, *Rec* tends to be relatively low. When *Rec* is high, *Pre* tends to be relatively low. To better evaluate the performance of the classifier, *F*1-Score is introduced to measure the comprehensive performance of the classifier. *F*1-Score is a commonly used measure for classification problems, which is the reconciled average of precision and recall. As shown in Equation (16), the *F*1-Score is the maximum of 1 and the minimum of 0. The higher the *F*1-Score, the higher it means that the values of *Pre* and *Rec* can reach the highest at the same time.

(16) $$F1=\frac{2\cdot Pre\cdot Rec}{Pre+Rec}\times 100\%$$

## 3. Results and Discussions

This study uses the Pytorch framework to verify the model and discusses the results under the Windows 11 system. The hardware configuration used in the experiment includes the 11th Gen Intel (R) Core (TM) i7-11800H@2.30 GHz processor, the main frequency is 2.30 GHz, the hard disk is 1 T, the memory is 16.0 GB, and the graphics card is Nvidia GTX 3050 model. The software environment includes Python 3.10, Pytorch1.11.0, and Cuda 11.3.

### 3.1. Model Construction and Selection of Hyperparameters

**Figure 9:**
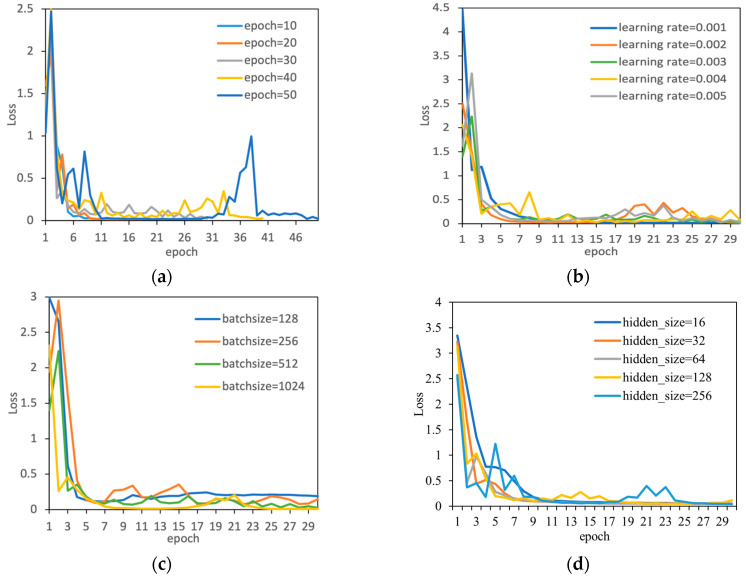
Comparison of model losses with different: (**a**) epochs, (**b**) learning rates, (**c**) batch sizes and (**d**) hidden_size.

In the process of model construction, hyperparameter tuning is carried out first. Hyperparameters are parameters manually set before the model starts training and cannot be automatically adjusted through model training. To obtain the best combination of hyperparameters, we designed three sets of comparison experiments, including epoch, learning rate, and batch size. Model Loss was used to evaluate the experimental results. Taking the CNN–LSTM–A model as an example, the comparison of experimental results is shown in Figure 9. All experiments used the same training and test sets.

#### 3.1.1. Epoch

In the comparison experiment, different epochs (10, 20, 30, 40, 50) and the same hyperparameters (learning rate = 0.003, batch size = 512) were used. Figure 9a shows the relationship between epoch and loss of the CNN–LSTM–A model on the test set. The comparison results show that the CNN–LSTM–A model proposed in this paper converges within 12–15 epochs, remains unchanged within 16–30 epochs, and starts to overfit within 30–40 epochs but still maintains good performance. The performance superiority of the proposed model and its adaptability to the dataset are proven.

#### 3.1.2. Learning Rate

Analyzed the different vectors (0.001, 0.002, 0.003, 0.004, 0.005) affect the performance of each model. The relationship between the learning rate and loss of the CNN–LSTM–A model proposed in this paper on the test set is shown in Figure 9b. When the learning rate is 0.002, the model converges the fastest, but overfitting occurs in the later stage. When the learning rate is 0.003, the model converges quickly and stably.

#### 3.1.3. Batch Size

The effects of different batch sizes (128, 256, 512, 1024) on the performance of each model were analyzed. The relationship between the learning rate and loss of the CNN–LSTM–A model proposed in this paper for different batch sizes on the test set is shown in Figure 9c. The error is larger when the batch size is less than 512, and the performance is better when the batch size is between 512 and 1024.

#### 3.1.4. Hidden Layer Size

Analyzed the impact of different hidden layer sizes (16, 32, 64, 128, 256) on the performance of each model. The relationship between the learning rate and loss of the CNN–LSTM–A model proposed in this paper for different hidden layer sizes on the test set is shown in Figure 9d. It shows a gradual convergence from a hidden layer size of 16 to 64, and a sudden change from a hidden layer size of 128 to 256.

Considering the rationality, rigor, and accuracy, this paper maintains the consistency of the hyperparameters between the proposed model and the benchmark model. The final hyperparameters of each model are shown in Table 4.

**Figure 10:**
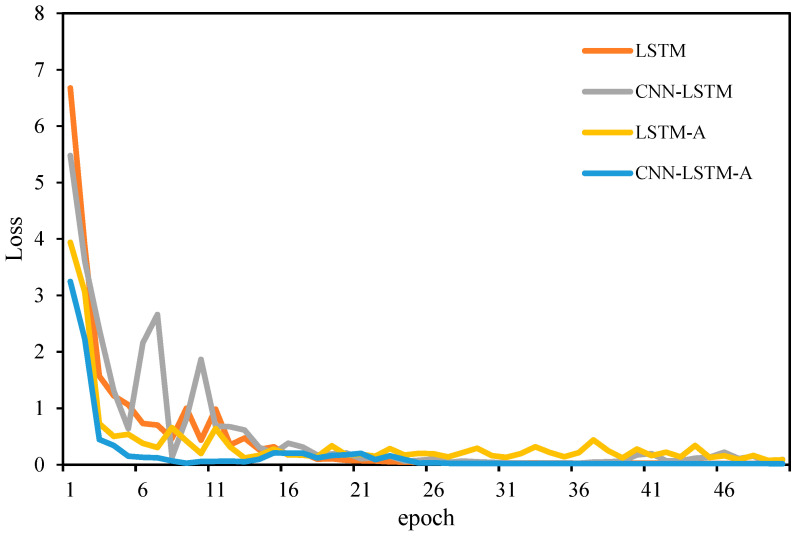
Fitness curves of different models.

In order to verify the validity of the proposed model, this study conducted a comparison experiment on the classification prediction effect of different models. The CNN–LSTM–A model is compared with an LSTM model, an LSTM–A model and a CNN–LSTM model. The faster the convergence speed of the algorithm, the better the performance of the prediction model. According to the above modeling process, the fitness curve of algorithm training is shown in Figure 10.

### 3.2. Comparative Experiments of Different Models

The prediction effect of the LSTM model, LSTM–A model, and CNN–LSTM on grain mildew grade was compared in the same dataset based on dataset 1 to verify the superiority of the CNN–LSTM–A model. The predicted value of the final mildew grade is rounded by rounding the decimal point. The comparison of prediction accuracy rates of different models is depicted in Table 5. The CNN–LSTM–A algorithm is the best in accuracy rate, accuracy rate, recall rate, and F1-score index.

**Figure 11:**
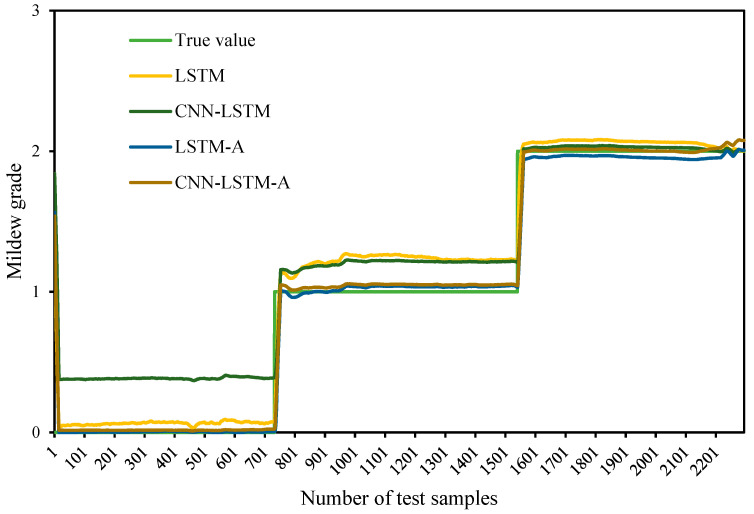
Prediction results of different models.

According to the experimental grain situation data, the food security situation can be predicted by referring to the safety evaluation level of the stored grain, to prepare food security control in advance. By comparing the forecast output values of the four prediction models, the CNN–LSTM–A model is more accurate in the whole prediction, and the prediction comparison diagram of the different models is shown in Figure 11. The X-axis represents the different test samples. The Y-axis represents the predicted value of mildew grade corresponding to the sample. By comparing the predicted values of the different models, the predicted values of the CNN–LSTM–A model were closer to the real values at the change junction of different mildew grades. Combined with the advantage analysis of the different models, the CNN–LSTM–A model combines the advantages of CNN, LSTM and Attention, fully mines time series data, and can effectively improve the accuracy of prediction.

### 3.3. Comparative Experiment of Dataset Training

Four independent datasets were constructed synchronously for comparative model training and test verification to verify the rationality and accuracy of the CNN–LSTM–A model training. The parameters and iteration times of each model were the same. The prediction accuracy of mildew grade in different datasets is similar, with the lowest accuracy being 98.48% and the highest 98.74%. The test accuracy and related parameters are shown in Table 6.

**Figure 12:**
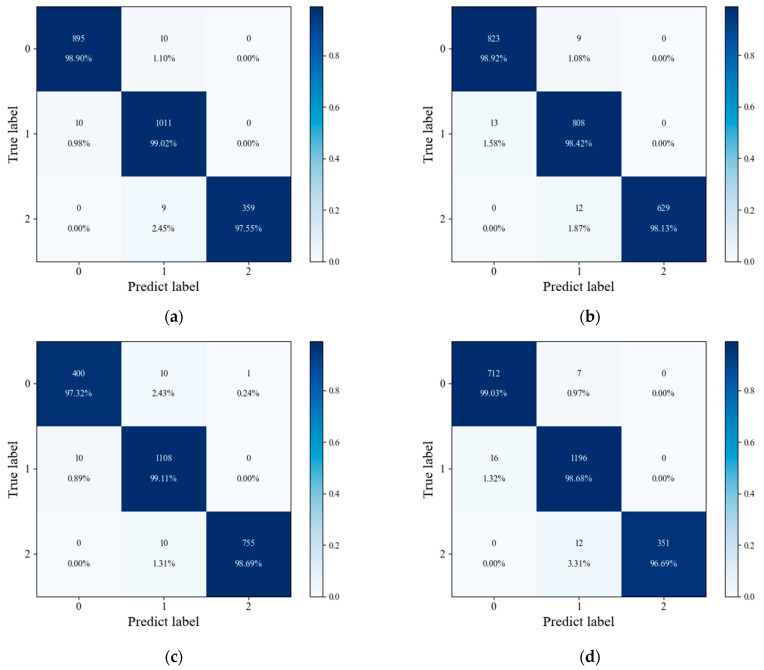
Comparison of model prediction confusion matrices under different datasets: (**a**) dataset 2, (**b**) dataset 3, (**c**) dataset 4, and (**d**) dataset 5.

After training the model with the datasets from the different experiments, the corresponding confusion matrix of each prediction result is shown in Figure 12.

**Figure 13:**
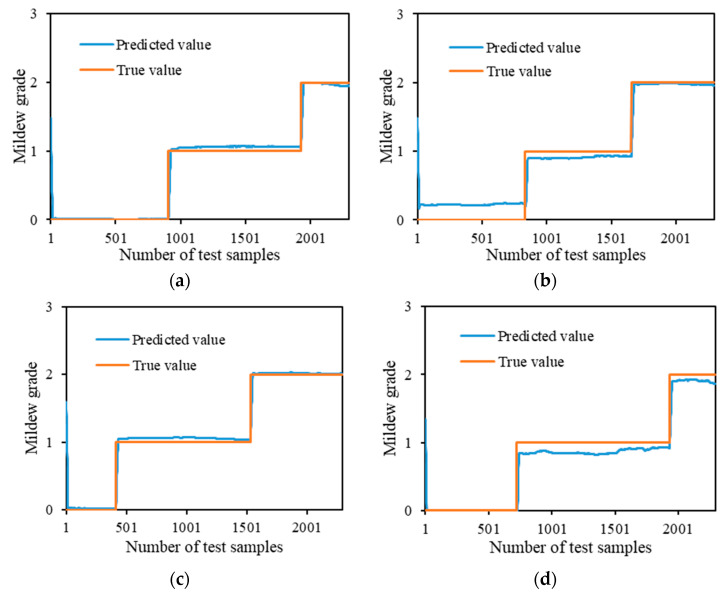
Comparison of model prediction results under different datasets: (**a**) dataset 2, (**b**) dataset 3, (**c**) dataset 4, and (**d**) dataset 5.

The experimental results show that the accuracy, precision, recall, and *F*1-score are stable in a certain range in different experimental datasets. The results showed that the CNN–LSTM–A prediction model had a good effect on the prediction of mildew grade. The comparison between the predicted value and the label value of the model under different datasets showed that the error rate was higher at the beginning of the test set and the transition of mildew grade, and the prediction result of the conventional process was more accurate. The specific comparison results are shown in Figure 13.

### 3.4. Analysis of Model Generalization

Datasets 2, 3, 4, and 5 are tested and analyzed after model training with dataset 1 to verify the generalization of the proposed model. The differences between the datasets (Table 3) are mainly reflected in the temperature and humidity of the environment. The average ambient temperature and humidity of dataset 1 were 8.25 °C and 60.46%RH, respectively, while the average temperature and humidity of the other datasets increased gradually. Using the CNN–LSTM–A prediction model, the accuracy of the test on these four test sets is relatively stable, exceeding 99.8%. The specific performance is shown in Table 7.

**Figure 14:**
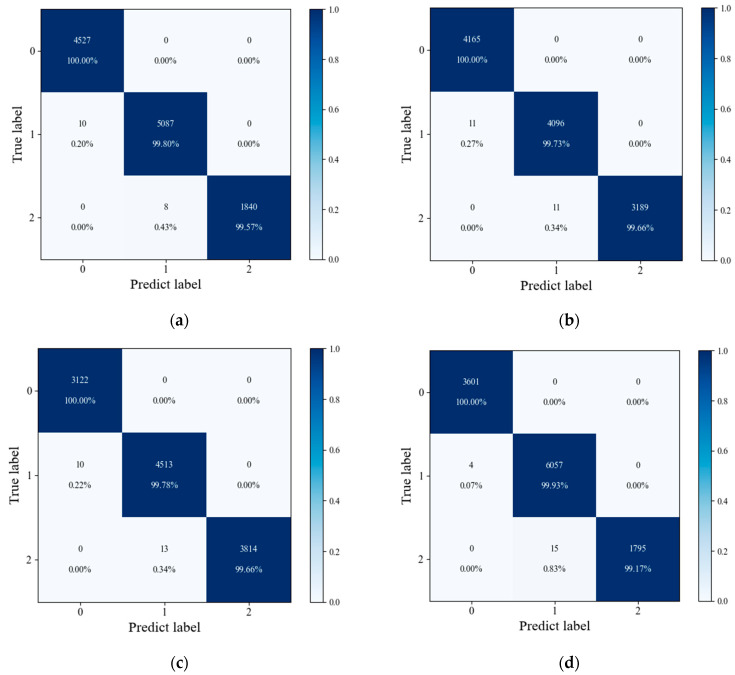
Comparison of model prediction confusion matrices under different test sets: (**a**) test set 2, (**b**) test set 3, (**c**) test set 4, and (**d**) test set 5.

The confusion matrix of predicted results from datasets of different experiments is shown in Figure 14.

**Figure 15:**
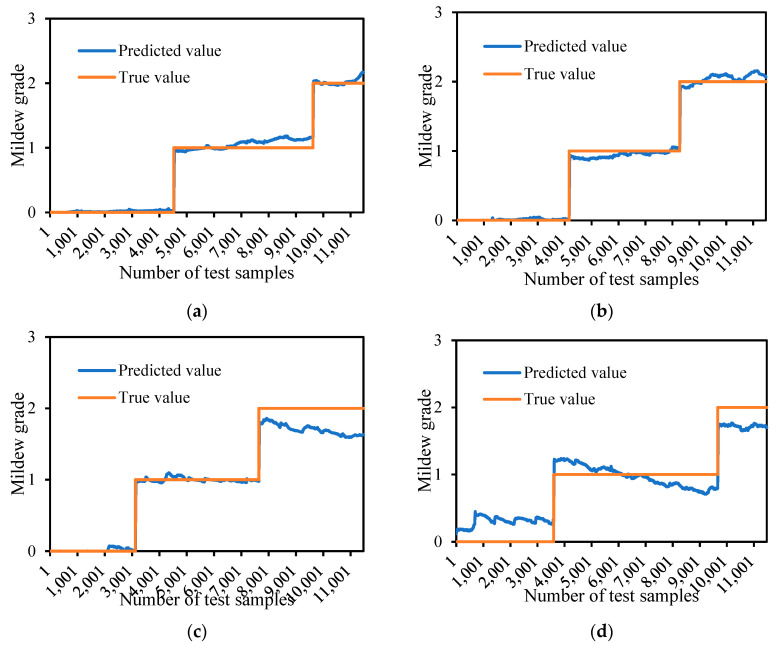
Comparison of model prediction results under different test sets: (**a**) test set 2, (**b**) test set 3, (**c**) test set 4, and (**d**) test set 5.

The experimental results show that the performance of the proposed model is relatively stable on different datasets. Combined with the comparative analysis of the predicted value and label value of the prediction model, based on the difference of storage temperature and humidity of different experiments (datasets 2, 3, 4, 5) and the storage temperature and humidity of the original training model (dataset 1), the closer the storage temperature of the original training model, the predicted value is relatively more stable. The prediction comparison results of models under different test datasets are shown in Figure 15.

The CNN–LSTM–A model proposed in this paper can flexibly process time series data of different lengths and can better capture the long-term dependence relationship in time series, to achieve higher prediction accuracy in time series prediction tasks. Compared with traditional models such as LSTM, the prediction accuracy is higher, which reflects the perfect effectiveness of the proposed model. At the same time, the model provides a new way to predict mildew in actual grain storage. The mildew prediction method can predict the rice mildew probability of granary, which can provide data reference for granary managers. When the probability of mildew is high, the manager can pay more attention to the grain warehouse. Thus, it is beneficial for the management of granaries, reducing the occurrence of grain mildew, and avoiding large-scale mildew to reduce the loss of grain enterprises.

## 4. Conclusions

Deep learning technology has not yet been maturely applied in the prediction and determination of grain mildew. This paper designed and developed a grain mildew monitoring device to monitor the micro-environmental parameters and image data during the grain mildew process to obtain high-quality experimental data and better predict the level of grain mildew. Using Zhejiang “Yongyou 15” rice as the research object, a simulated storage mildew experiment was designed. Environmental information about the stored grain was obtained through integrated information sensing equipment, and the mildew status of the rice was captured by a camera, recording the changes in mildew condition. Meanwhile, professional technicians labeled the mildew grades based on mildew images, constructing a multimodal dataset of the rice mildew process.

The CNN–LSTM–A model was used to train the rice mildew grades effectively. Compared with other experimental models, this model’s prediction accuracy for mildew grades improved by 0.96%, 0.43%, and 0.13% over the LSTM and other models, respectively, and the F1-score improved by 0.95%, 0.44%, and 0.13%, respectively. Additionally, the CNN–LSTM–A model was used to train and validate the constructed four independent datasets. Comparative analysis showed that the accuracy was stable above 98%, indicating high stability of the model. For the generalization analysis of the model, four experimental datasets from different environments were tested and validated, with accuracy reaching 99.8%. Analysis revealed that the closer the temperature and humidity environment of the test data were to the training dataset, the smaller the deviation between the predicted value and the actual value. The greater the deviation in temperature and humidity from the training dataset, the greater the deviation between the predicted value and the actual value. Therefore, the CNN–LSTM–A-based rice mildew grade prediction model proposed in this paper has high prediction accuracy and good generalization performance.

The results of this study provide a technical method for the early prediction of mildew grades during grain storage, which is beneficial for the prevention and control of rice mildew in the storage process. However, there are certain limitations to the study. The types of rice varieties and the number of mildew experiments in this study were insufficient, and the classification of rice mildew types was not realized. In future studies, more comprehensive mildew datasets should be developed, such as introducing rice varieties from different major rice-producing areas such as Jiangsu and setting environmental conditions such as temperature and humidity in a wider range. Additionally, automatic identification and image labeling technologies, such as low-cost RGB cameras and computer vision technology, should be employed to improve the efficiency of mildew image labeling and the precision of mildew grading. Further optimization of the prediction model should be conducted continuously using the dataset to enhance both accuracy and robustness. As deep learning technology advances, the latest prediction models, such as Transformer and Informer, could be explored in rice mildew prediction to enhance training efficiency, reduce resource consumption, improve predictive performance, and manage longer time series or multimodal data.

## Figures and Tables

**Table 1:** Sensors and their parameters of the grain mildew process monitoring system.

Transducers	Measuring Range	Measurement Accuracy	Manufacturer
Temperature and humidity sensors	−40~100 °C; 0~100%RH	Temperature: ≤±0.5 °C Humidity: ≤±3% RH	Weihai JXCT Electronics Co., Ltd., Weihai, China
Grain temperature and moisture sensors	−40~100 °C; 0~60%RH	Temperature: ≤±0.5 °C Humidity: ≤±1% RH	Weihai JXCT Electronics Co., Ltd., Weihai, China
Carbon dioxide sensor	0~50,000 ppm	±5% F.S (25 °C)	Weihai JXCT Electronics Co., Ltd., Weihai, China
Oxygen Sensor	0~30%	<±3% of reading (25 °C)	Weihai JXCT Electronics Co., Ltd., Weihai, China

**Table 2:** Description of different experimental data sets.

Datasets	Experimental Time	Data	Average Ambient Temperature	Average Ambient Humidity
1	29 January 2023~6 February 2023	11,492	8.25 °C	60.46% RH
2	1 March 2023~9 March 2023	11,492	13.76 °C	57.97% RH
3	26 March 2023~3 April 2023	11,492	14.85 °C	63.13% RH
4	17 April 2023~25 April 2023	11,492	19.88 °C	61.85% RH
5	3 May 2023~11 May 2023	11,492	21.16 °C	62.48% RH

**Table 3:** Grain storage safety assessment.

Grade	Safety Assessment	Main Growing Fungus	Color and Luster
0	Safe	Essentially no harm to fungal growth	Normalcy
1	Critical (critical control area)	Gray-green Aspergillus predominantly, a small amount of white Aspergillus and other growth will occur later on	Grayish color with no luster
2	Harmful	Aspergillus gray-green growth is gradually replaced by Aspergillus albicans, and a small amount of other fungal growth occurs	Darkened color or brownish shell

**Table 4:** Model hyper-parameters.

Hyper-Parameter	LSTM	CNN + LSTM	LSTM + Attention	CNN + LSTM + Attention
Number of hidden layers	64	64	64	64
Lot size	512	512	512	512
Dropout	\	\	0.8	0.8
Optimizer	AdamW	AdamW	AdamW	AdamW
Training period (epoch)	50	50	50	50
Initial learning rate	0.0003	0.0003	0.0003	0.0003
Loss function	MSELoss	MSELoss	MSELoss	MSELoss

**Table 5:** Comparison of evaluation indicators for each model.

Modeling Algorithm	Number of Test Samples	Correct Number of Samples	*Acc*	*Pre*	*Rec*	*F*1
LSTM	2294	2242	97.73%	97.97%	97.69%	97.76%
CNN–LSTM	2294	2254	98.26%	98.28%	98.26%	98.27%
LSTM–A	2294	2261	98.56%	98.59%	98.56%	98.58%
CNN–LSTM–A	2294	2264	98.69%	98.72%	98.67%	98.71%

**Table 6:** Classification accuracy for different experiments.

Dataset	Number of Test Samples	Number of Correct Samples	*Acc*	*Pre*	*Rec*	*F*1
2	2294	2265	98.74%	98.74%	98.49%	98.74%
3	2294	2260	98.52%	98.52%	98.49%	98.56%
4	2294	2263	98.65	98.65%	98.37%	98.46%
5	2294	2259	98.47%	98.48%	98.13%	98.43%

**Table 7:** Classification accuracy for different test sets.

Test Dataset	Number of Test Samples	Number of Correct Samples	*Acc*	*Pre*	*Rec*	*F*1
2	11,492	11,454	99.84%	99.84%	99.83%	99.79%
3	11,492	11,450	99.81%	99.81%	99.81%	99.80%
4	11,492	11,449	99.80%	99.80%	99.81%	99.81%
5	11,492	11,453	99.83%	99.83%	99.80%	99.70%

## Data Availability

The raw data supporting the conclusions of this article will be made available by the authors upon request.
